# The Effect of Surface Pretreatments on the Bond Strength of Hybrid CAD/CAM with Composite Elevation

**DOI:** 10.3390/jfb17030157

**Published:** 2026-03-23

**Authors:** Mehmet Ali Fildisi, Burcu Oglakci Ozkoc, Zumrut Ceren Ozduman, Evrim Eliguzeloglu Dalkilic

**Affiliations:** 1 Department of Restorative Dentistry, Faculty of Dentistry, University of Health Sciences, Istanbul 34668, Turkey; 2 Department of Restorative Dentistry, Faculty of Dentistry, Bezmialem Vakif University, Istanbul 34093, Turkey

**Keywords:** computer-aided design, computer-aided manufacturing, hydrofluoric acid, air abrasion, dental, composite resins, dental bonding

## Abstract

In computer-aided design/computer-aided manufacturing (CAD/CAM) restorations for severely damaged teeth, the cavity floor or proximal margins may be elevated with composite resin to improve adhesion. This in vitro study investigated how different surface pretreatment methods affect the shear bond strength (SBS) of hybrid CAD/CAM materials to dentin or composite surfaces, simulating clinical situations of composite elevation. Hybrid CAD/CAM samples were bonded to dentin or composite substrates following different surface pretreatment protocols and cemented using a dual-cure adhesive resin cement. The samples were thermocycled and subjected to shear bond strength testing, and failure modes were analyzed. The SBS in the sandblasting (SB)+Dentin group and hydrofluoric acid (HF)+Dentin was significantly higher than that in the SB+Composite and HF+Composite groups (*p* < 0.05). Untreated+composite and untreated+dentin groups showed significantly lower SBS (*p* < 0.05). Failure mode analysis revealed a predominance of cohesive failures in the SB+Dentin group, while adhesive failures were more frequently observed in most of the other groups. SB-treated and HF-etched hybrid CAD/CAM materials showed more favorable bonding behavior to dentin than to composite, highlighting that bonding to the elevated composite layer may be less effective than bonding directly to prepared dentin.

## 1. Introduction

Failures in both conservative and traditional prosthetic dentistry, particularly in cases involving deep cervical lesions and subgingival margins, remain a significant clinical concern due to the risk of decementation and recurrent caries. Innovative technologies to address these challenges have been increasingly incorporated into conservative and restorative dental approaches. Within conservative dentistry, modern methods such as laser applications for caries prevention and tissue preservation have been reported, whereas in restorative dentistry, the increasing integration of digital technologies has led to the increased use of computer-aided design and computer-aided manufacturing (CAD/CAM) restorations in clinical practice [1,2].

CAD/CAM technologies enable the development of stable esthetic materials, improved adaptation to restorations, and more time-efficient fabrication of indirect restorations. Hybrid CAD/CAM materials are preferred as they are easier to produce, less abrasive to opposing teeth, and have a modulus of elasticity closer to that of dentin. In addition, unlike ceramic-based restorative materials, hybrid CAD/CAM materials can be modified and repaired with composite resins [3].

There is no consensus in the literature regarding the restoration of posterior teeth with large defects [4]. The traditional method involves placing full crowns, including a post and core structure [5]; however, the development of adhesive techniques has recently led to the increased adoption of clinical approaches such as inlay, onlay, overlay, and endocrown CAD/CAM restorations. Compared with direct adhesive techniques, CAD/CAM restorations demonstrate several notable advantages—particularly in extensive cavities—including refined anatomical morphology, optimized contour, enhanced fracture resistance, and improved wear performance [6]. In addition, unlike post-and-core crown restorations, adhesively bonded indirect restorations can minimize preparation and preserve tooth structure in cases of extensive tooth tissue loss with large defects.

In the context of CAD/CAM restorations for severely damaged teeth, especially those extending below the cementoenamel junction, indirect restorations with composite resins in the proximal regions may be necessary following cavity shaping and cervical margin relocation (deep margin elevation). Moreover, in some cases, particularly for endocrowns, cavities should be reshaped and elevated with composite resins to manage the cavity design, especially in the pulp chamber region, and improve the adhesion of CAD/CAM restorations for root canal-treated teeth with large defects [7].

Successful adhesion between an indirect restoration and resin cement increases the resistance of either the tooth or the restoration and provides better retention, marginal adaptation, and sealing. Thus, surface pretreatment is necessary for optimal adhesion between resin cement and CAD/CAM restorations. With increased contact area, surface energy, and wettability, resin cement can better infiltrate the surfaces of CAD/CAM materials and, consequently, undergo micromechanical locking [8]. Various surface treatments, such as sandblasting (SB) with aluminum oxide (Al_2_O_3_) particles and chemical etching with hydrofluoric acid (HF), have been proposed for hybrid CAD/CAM materials. HF interacts with silica, dissolving the glass phase of a hybrid CAD/CAM material and increasing its surface roughness and bond strength [9]. The SB process removes contaminated layers and oxides, revealing the active, clean surface of the restorative material and enabling micromechanical bonding [10].

Limited literature is available on the adhesion of hybrid CAD/CAM materials using composite elevation and various surface treatments [11]. Accordingly, this in vitro study was conducted to evaluate the influence of various surface pretreatment protocols on the shear bond strength (SBS) of hybrid CAD/CAM materials to dentin or composite substrates. The first null hypothesis was that the type of surface pretreatment would have no significant effect on the SBS of the hybrid CAD/CAM material to either dentin or composite surfaces. The second null hypothesis was that composite elevation would not affect the SBS of hybrid CAD/CAM materials with different surface pretreatments.

## 2. Materials and Methods

This in vitro study was conducted in accordance with the tenets of the Declaration of Helsinki and was approved by the Ethics Committee of Bezmialem Vakif University (Process number: 2024/353). Informed consent was obtained from all participants or their legal guardians prior to the use of any extracted human teeth in this study.

### 2.1. Sample Size Calculation

The sample size was determined according to the expected effect size between the two groups, as described in the relevant literature [12]. For the SBS test, a per-group sample size of 15 was needed to attain a medium effect size (d = 0.50) with 90% power and a type 1 error rate of 5%.

### 2.2. Study Design and Experimental Groups

**Figure 1:**
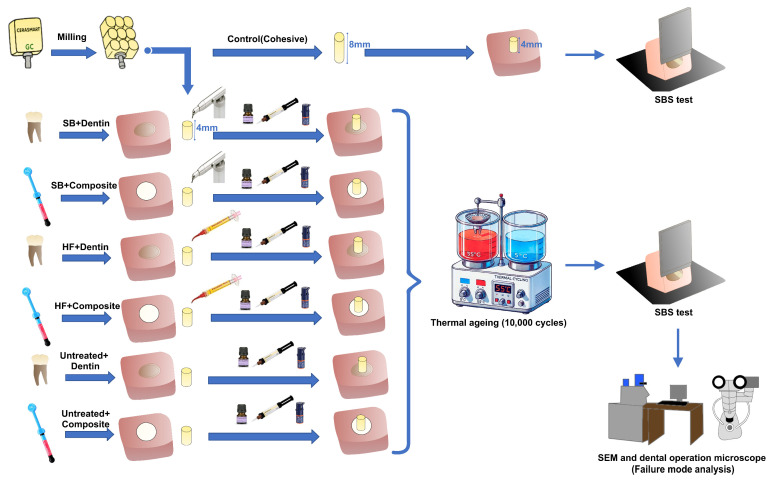
A diagram of the study and schematic representation of the experimental groups. SB: sandblasting; HF: hydrofluoric acid, SEM: scanning electron microscope, SBS: shear bond strength, mm: millimeter.

A diagram of the experimental procedure is presented in Figure 1. The restorative materials used in this study, along with their brand names, manufacturers, lot numbers, and chemical compositions, are shown in Table 1.

This study assessed two different variables:

(1) Adhesion substrates:

Dentin groups: No composite elevation simulation.

Composite groups: Composite elevation simulation.

(2) Surface treatment procedures for hybrid CAD/CAM materials: Untreated, SB or HF.

A total of 90 samples were prepared, comprising six experimental groups according to the adhesion substrates (dentin or composite) and surface pretreatments (Untreated, SB or HF) (*n* = 15). Additionally, 15 samples were used as a control (cohesive) group (*n* = 15). The specific groups were as follows:

SB+Dentin: SB-treated hybrid CAD/CAM+Dentin.

SB+Composite: SB-treated hybrid CAD/CAM+Composite.

HF+Dentin: HF-treated hybrid CAD/CAM+Dentin.

HF+Composite: HF-treated hybrid CAD/CAM+Composite.

Untreated+Dentin: No surface pretreated hybrid CAD/CAM+Dentin.

Untreated+Composite: No surface pretreated hybrid CAD/CAM+Composite.

Control (cohesive): Hybrid CAD/CAM cylinder.

### 2.3. Preparation of Dentin Groups

In total, 30 extracted sound human lower molars were collected from the SB and HF groups (*n* = 15 per group). The molars were stored in saline at room temperature and utilized for experimentation within 3 months of extraction. Using a water-cooled model trimmer (MT3 Wet Trimmer, Renfert GmbH, Hilzingen, Germany), the occlusal one-third of the crown was cut parallel to the horizontal plane. The exposed midcoronal dentin was polished with 600-grit silicon carbide paper on a metallographic polisher (Minitech 233, Presi, Grenoble, France) with water cooling for 60 s to achieve a standardized smear layer. The teeth were examined under a stereomicroscope (SMZ 1000, Nikon, Tokyo, Japan) to detect the presence of enamel or pulp exposure.

### 2.4. Preparation of Composite Groups

A total of 30 cylindrical-shaped composite resin samples were prepared with a conventional composite resin (diameter: 5 mm, height: 4 mm) (Gradia Direct Posterior, GC Corp, Tokyo, Japan) using a plastic mold for the two experimental groups (SB and HF; *n* = 15 per group). The mold was positioned on glass microscope slides. Composite resin layers (2 mm thick) were placed in the mold incrementally. A transparent mylar matrix strip was placed over the resin surface and firm pressure was applied with a dental spatula to remove excess material and achieve a smooth surface before light curing. Each layer was polymerized with a light-emitting diode light curing unit (LED LCU; Valo, Ultradent, South Jordan, UT, USA) for 20 s at 1000 mW/cm^2^.

Then, for all four experimental groups (dentin and composite groups), the middle-coronal dentin and composite resin samples were embedded in acrylic resin blocks (15 × 15 × 10 mm^3^) to expose the superficial surfaces.

### 2.5. Hybrid CAD/CAM Material Preparation

For all six experimental groups, a total of 90 cylinder-shaped hybrid CAD/CAM blocks (diameter: 3 mm, height: 4 mm) (Cerasmart, GC Corp, Tokyo, Japan) were obtained with a CAD/CAM milling system (CEREC SW 4.6, Sirona Dental Systems, Bensheim, Germany). Additionally, for the control group, 15 cylinder-shaped hybrid CAD/CAM blocks (diameter: 3 mm, height: 8 mm) were prepared as described above. For the control group, the hybrid CAD/CAM samples were embedded at a height of 4 mm using acrylic resin.

### 2.6. Surface Pretreatment Procedures

Untreatment: For the experimental groups, hybrid CAD/CAM samples were prepared as described above. No surface pretreatment procedure was performed.

SB pretreatment: For the experimental groups, hybrid CAD/CAM samples were prepared as described above, and their luting surfaces were pretreated with the SB method using 50 µm Al_2_O_3_ particles at 2 bar pressure with a 10 mm distance for 10 s. The residues were then cleaned in an ultrasonic bath with isopropyl alcohol for 3 min and air-dried.

HF pretreatment: For the experimental groups, hybrid CAD/CAM samples were prepared as described above, and their luting surfaces were pretreated with HF (9%) (Ultradent Porcelain Etch, Ultradent Products Inc., South Jordan, UT, USA) for 60 s, then rinsed with water for 20 s according to the manufacturer’s instructions. The samples were then air-dried.

### 2.7. Cementation

Following pretreatment of the CAD/CAM surfaces, the silane coupling agent (Monobond Plus, Ivoclar Vivadent AG, Schaan, Liechtenstein) was applied to the surfaces with a microbrush for 60 s and then air-dried for 30 s. Subsequently, for adhesion substrates (composite and dentin), a universal adhesive system (Single Bond Universal Adhesive, 3M ESPE, St. Paul, MN, USA) was applied with a microbrush for 20 s, and the sample was gently air-dried. Dual-cure adhesive resin cement RelyX Ultimate (3M ESPE, St. Paul, MN, USA) was applied for the cementation of ceramic cylinders and polymerized with an LED light-curing unit (LCU; irradiance of 1000 mW/cm^2^). The samples were light-cured for 20 s from each side to ensure optimal polymerization. The intensity of the LCU was controlled using a radiometer throughout the experiment (Demetron LED Radiometer, Kerr Corp. Orange, CA, USA). All the samples were stored in distilled water at 37 °C for 24 h. Then, the samples were subjected to 10,000 thermocycles (5–55 °C; dwell time: 30 s; and transfer time: 10 s) in a thermocycler (SD Mechatronic Thermocycler, Westerham, Germany), corresponding to one year of clinical use. All restorative procedures were performed by a single operator, following the manufacturer’s instructions [13,14].

### 2.8. SBS Test

All the samples were subjected to SBS testing using a universal testing machine (AGS-X, Shimadzu Corp., Kyoto, Japan) with a crosshead speed of 1 mm/min. The testing load was directly applied to the interface where the CAD/CAM cylinder was cemented to dentin or composite surfaces or to the region where it was embedded in acrylic resin (in the control group) until fracture with a knife-edged chisel [15]. The SBS (MPa) was calculated by dividing the maximum force recorded at failure by the bonded surface area. The values were recorded in Newtons and converted to MPa.

### 2.9. Failure Mode Analysis

A single operator determined the mode of failure in the fractured samples using a dental operation microscope (Labomed Magna, Labo America, Inc., Fremont, CA, USA) at 15× magnification. Additionally, representative samples were selected and evaluated by scanning electron microscope (SEM) (Evo LS10, Zeiss, Oberkochen, Germany). The samples were subjected to gold sputter coating in secondary mode at accelerating voltages of 10–30 kV. The images were captured at 50× magnification.

The failure modes were recorded and classified as adhesive failure at the CAD/CAM–luting resin cement interface, adhesive failure at the dentin/composite–luting resin cement interface, cohesive failure, or mixed failure.

The cohesive failure mode was considered if the fracture occurred in the CAD/CAM material, dentin/composite, or luting resin cement. The mixed failure mode was considered if the fracture occurred in both the CAD/CAM material and the composite resin/dentin or luting resin cement and along the junction between the CAD/CAM material and the composite resin/dentin or luting resin cement.

### 2.10. Statistical Analysis

Statistical analyses were conducted using SPSS 22.0 for Windows (SPSS Inc., Chicago, IL, USA). The Shapiro–Wilk test was initially conducted to assess the normality of the variables, followed by an analysis of the homogeneity of variances using Levene’s test. The data were found to be normally distributed. A two-way ANOVA was conducted to compare differences within and between groups, with Bonferroni post hoc testing applied to all pairwise comparisons for SBS data. Chi-square analysis was also performed for failure mode analysis. A confidence level of 0.05 was considered to indicate significance in all analyses. Effect size indices (η^2^) were also used to quantify the magnitude of group differences.

## 3. Results

### 3.1. SBS Test Results

**Figure 2:**
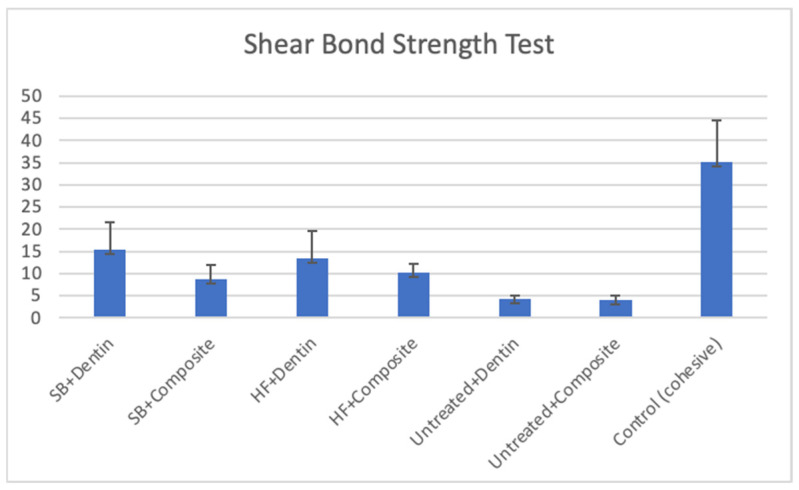
Mean shear bond strength values (MPa) for each group.

The mean SBS values and standard deviations (±SDs) of all the tested groups are shown in Table 2 and Figure 2.

Two-way ANOVA revealed that a significant interaction between surface treatment and substrate was detected (*p* = 0.041, η^2^ = 0.087). Substrate type explained a substantial proportion of the variance in SBS, with η^2^ values of 0.357 for dentin and 0.132 for composite. The surface treatment method also showed a statistically significant effect (*p* < 0.05), although with a smaller effect size (η^2^ ranging from 0.057 to 0.204).

The highest SBS values were observed in the cohesive (control) group (*p* < 0.05). Regarding the type of adhesion substrate, the SB+Dentin and HF+Dentin groups demonstrated significantly greater SBS than the SB+Composite and HF+Composite groups (*p* < 0.05). The untreated dentin group exhibited similar SBS values to the untreated composite group (*p* > 0.05). Regarding the surface pretreatment methods, no significant differences in SBS were found between the SB+Dentin and HF+Dentin groups or between the SB+Composite and HF+Composite groups (*p* > 0.05) (Table 2, Figure 2). Untreated dentin and composite groups showed significantly lower SBS values than other pretreated dentin and composite groups (*p* < 0.05) (Table 2) (Figure 2).

### 3.2. Failure Mode Analysis Results

**Figure 3:**
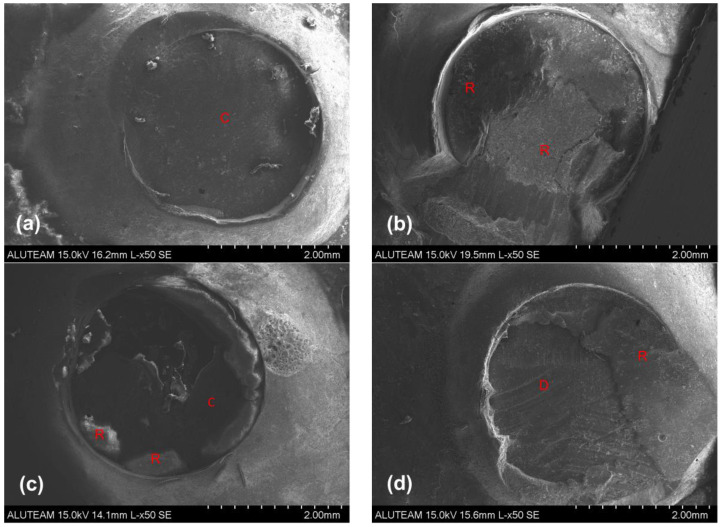
Representative scanning electron microscopy images of failure modes: (**a**) SB+Composite (adhesive failure), (**b**) SB+Dentin (cohesive failure), (**c**) HF+Dentin (mix failure), (**d**) HF+Composite (mix failure). R: resin cement; D: dentin, C: composite.

The failure modes of the fractured surfaces for all the tested groups are shown in Table 3. Representative SEM images of each group are presented in Figure 3. In the dental operating microscope analysis, the predominant failure mode was adhesive failure at the dentin/composite–resin cement interface (Figure 3a) in the HF+Composite, HF+Dentin, SB+Composite, Untreated+Dentin, and Untreated+Composite groups. In contrast, the predominant failure mode in the SB+Dentin group was cohesive failure occurring exclusively within the resin cement (Figure 3b).

The distribution of failure modes differed significantly among the groups (*p* < 0.001, Table 3). According to the chi-square test, the distribution of failure modes showed a statistically significant difference among the groups (*p* < 0.001). The proportion of adhesive failures in the SB-dentin group was significantly lower than those observed in the untreated dentin, untreated composite, and HF-composite groups.

No groups exhibited adhesive failure at the CAD/CAM material–resin cement interface. No cohesive failures were detected in the HF+Composite, HF+Dentin, Untreated+Dentin, and Untreated+Composite groups. Mixed failure was the second most frequently observed failure type. Representative fractured composite surfaces (Figure 3c) and images of exposed dentin (Figure 3d) in mixed failure samples are shown. No fractures were observed within the CAD/CAM material, composite, or dentin substrate in any samples.

## 4. Discussion

Composite elevation is frequently applied in clinical practice to improve isolation, enhance impression accuracy, and facilitate cementation by relocating deep margins coronally. Bonding of indirect restoration materials to composite rather than dentin is an important area of investigation in adhesive dentistry; however, the available literature on this topic remains limited [16,17]. Thus, we conducted this in vitro study to investigate the effects of different surface pretreatment methods on the SBS of hybrid CAD/CAM materials to dentin and composite surfaces.

Surface roughening of CAD/CAM materials affects the performance of adhesive resin cements, thereby improving adhesion efficacy [11]. The surface topography resulting from SB depends on factors such as particle size, propulsion pressure, and application duration [18]. Muhammed et al. [19] reported that SB Cerasmart at 2 bar for 10 s markedly increased surface roughness. Furthermore, they noted that higher pressures led to surface degradation and reduced mechanical strength. Yoshihara et al. [20] reported improved surface roughness when SB was performed with 50 μm particles at 2 bar pressure. The SB procedure used in the current study was selected on the basis of these findings.

The HF etching technique aims to dissolve filler particles, creating a porous surface [21,22]. Muhammed et al. [19] reported that using 9% HF on Cerasmart and Lava Ultimate was effective for surface pretreatment. Straface et al. [23] evaluated different HF concentrations and application times on various CAD/CAM materials, revealing that 9% HF applied for 60 s was effective for the cementation of hybrid CAD/CAM materials. Thus, 9% HF was applied for 60 s to modify the surfaces of the hybrid CAD/CAM material in the present study.

Chemical bonding and micromechanical interlocks on the restoration surface are critical for the adhesion of indirect restorative materials [24]. Universal adhesive systems are available in multipurpose formulations and can be used to bond to both tooth tissues and metal, ceramic, and composite restorative materials [25]. The separate application of silane coupling agents increases the wettability of ceramic surfaces by forming chemical bonds between the inorganic and organic phases, thereby reinforcing adhesion [9]. Dual-cure adhesive resin cements have become widely used in indirect CAD/CAM restorations due to their ability to ensure reliable polymerization even in areas with limited light transmission. Accordingly, Monobond Plus was used as a silane coupling agent for CAD/CAM materials in this study. The universal adhesive system Single Bond Universal was subsequently applied to the CAD/CAM material and composite/dentin. Due to its widespread popularity in clinical applications, the dual-cure adhesive resin cement “RelyX Ultimate” was also selected for cementation [26,27].

Notably, the cohesive strengths of the CAD/CAM materials were also measured and included as a control group in this study. In contrast to the experimental groups, which were treated with 4 mm high CAD/CAM cylinders bonded to dentin or composite surfaces, the control group received 8 mm high CAD/CAM cylinders tested without bonding. This approach was employed to evaluate the intrinsic cohesive strength of the CAD/CAM material. A similar methodology was reported by Aquino et al. [28] and Oglakci et al. [29], who used 8 mm bulk-fill composite cylinders as a control group to assess cohesive strength in comparison with 4 mm bulk-fill composite bonded samples. Remarkably, the control group showed the highest SBS values in the present study.

In the present study, no surface pretreatment led to significantly lower SBS than both surface pretreatment methods for composite and dentin substrates. This finding further highlights the crucial role of surface pretreatment in achieving reliable adhesion, particularly when composite elevation is performed. Since surface pretreatments can increase surface roughness and surface energy, promoting micromechanical interlocking. In contrast, untreated surfaces remain relatively smooth and may retain contaminants, limiting adhesive penetration. Moreover, comparing the surface pretreatment methods, no significant differences in SBS between SB and HF etching were observed for either adhesion substrate. This finding may be attributed to the microstructure of the hybrid CAD/CAM material. Cerasmart is classified as a resin-nanoceramic composite, consisting of a highly cross-linked resin matrix reinforced with uniformly distributed silica and barium glass nanoparticles. Unlike glass-ceramics, where HF etching selectively dissolves the glassy phase and creates pronounced micromechanical retention, the polymer-rich matrix of resin-nanoceramic materials limits the etching capability of HF. Consequently, HF treatment may produce only limited surface roughening in this material. This may also be influenced by the concentration and application time of HF. On the other hand, excessively aggressive sandblasting (SB) protocols have been reported to potentially compromise bonding performance through surface damage or microcrack formation due to the resin-based matrix structure of hybrid CAD/CAM materials. This effect has been associated with air-abrasion parameters, particularly prolonged application time, suggesting that SB may produce different surface morphologies depending on the procedural conditions applied [8,30]. Sandblasting increases surface roughness through mechanical abrasion, generating micro-irregularities and increasing the available bonding surface area. Therefore, both surface treatments may create similar micromechanical interlocking potential, resulting in comparable bond strength values. Consistent with these findings, Chuenjit et al. [31] compared the effects of HF etching and air abrasion on the bond strength of self-adhesive resin cement to those of four different hybrid CAD/CAM materials (Block HC, Vita Enamic, Cerasmart, and Lava Ultimate) and reported no significant differences in the bond strength of Cerasmart between the two pretreatment methods.

However, contradictory results exist in the literature, such as the study by Mangoush et al. [10], which investigated the SBS of adhesive resin luting cement to Cerasmart 270 following HF and air abrasion protocols and demonstrated that air abrasion resulted in higher SBS values than acid etching. These differences may be partially attributable to the lower HF concentration (4.5%) used in the study by Mangoush et al. [10], which may have limited surface roughening and thereby insufficiently influenced bonding performance. Abdou et al. [32] reported that, compared with SB, HF etching provided superior bonding performance for hybrid CAD/CAM materials, including Vita Enamic to resin cement. These variations in bonding performance may result from differences in study design, luting protocols, and CAD/CAM material composition. Regarding material composition, comparing the ceramic content of hybrid CAD/CAM materials by weight, VITA Enamic contains 86%, Cerasmart contains 71%, and Lava Ultimate contains 80% [3]. In addition, VITA Enamic exhibits a dual-network structure formed by polymer infiltration into a feldspathic ceramic matrix, Cerasmart is characterized by a homogeneously distributed nanoceramic phase within a highly cross-linked resin matrix, and Lava Ultimate comprises nanoceramic clusters embedded in a highly cross-linked resin matrix [3,33,34]. These structural and compositional differences may influence the surface characteristics of the materials and their responses to pretreatment methods, potentially explaining variations in bond-strength outcomes across studies.

With advances in composite resin technology, the use of composite resins to increase deep cervical margins and/or support the residual tooth structure has become more widespread in cases of significant structural loss when an indirect restoration is planned. In the present study, we aimed to simulate the clinical conditions of elevating the pulp chamber or deep margins with a composite material prior to indirect restoration. In such scenarios, the adhesion of CAD/CAM restorations to composite surfaces becomes necessary. In our earlier study, hybrid CAD/CAM restorations with composite elevation on pulp chambers demonstrated greater fracture strength than those without composite elevation [7,16]. According to recent studies, composite elevation using highly filled flowable resin composites can be effectively used under ceramic inlays to facilitate isolation and cementation procedures while minimizing the stress concentration [17,35]. Kielbassa and Philipp’s [36] systematic review of proximal box elevation (PBE) demonstrated that various restorative materials achieved clinically acceptable outcomes via this technique, thereby enhancing the applicability and potentially contributing to the long-term clinical success and periodontal health of indirect restorations. Nevertheless, the researchers recommended that further studies be conducted on composite elevation. Isolan et al. [25] also investigated the effect of PBE on the bond strength of composite inlays, demonstrating that PBE increased bond strength. Notably, the present study was designed to examine scenarios in which the pulp chamber or the deep gingival floor at the proximal region of a tooth is elevated with a conventional composite. Therefore, the SBS of CAD/CAM indirect restorations to conventional composite or dentin surfaces was evaluated. Our findings revealed that, in the SB and HF etching groups, composite elevation resulted in a markedly lower SBS than without elevation (dentin substrate). This finding may be attributed to the additional bonding interface created by the composite layer, which can introduce potential interfacial defects. Moreover, polymerized composite surfaces often present reduced surface energy and fewer unreacted methacrylate groups, which may limit adhesive interaction and compromise bonding effectiveness compared with direct bonding to dentin.

Given these results, the first null hypothesis, that different surface pretreatments would not affect the SBS of hybrid CAD/CAM materials on dentin or composite surfaces, was accepted. No significant differences in SBS were found between SB and HF applications. The second null hypothesis, which proposed that composite elevation would not affect the SBS of hybrid CAD/CAM materials with different surface pretreatments, was rejected. The SBS of the SB and HF etching hybrid CAD/CAM material to dentin was significantly greater than that to composite surfaces.

**Figure 4:**
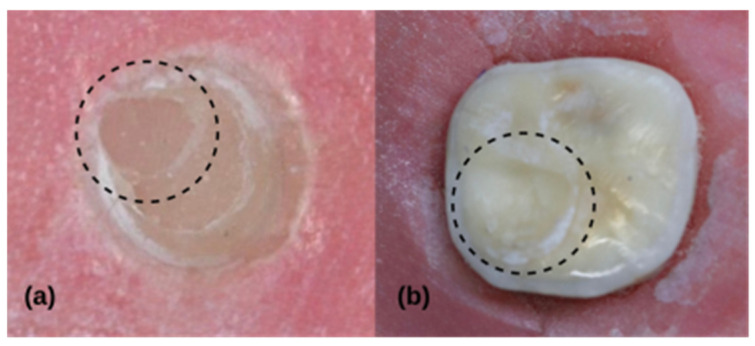
Dental operation microscope images of the failure modes: (**a**) SB+Composite (adhesive failure) and (**b**) SB+Dentin (cohesive failure). Circled areas denote the cementation zones.

In the failure mode analysis, adhesive failure was predominant in both the HF etching and untreated groups. Although the SB+Composite group also demonstrated a predominance of adhesive failures (Figure 4a), this pattern shifted in the SB+Dentin group, in which cohesive failures predominated (Figure 4b). In the SB+Dentin group, cohesive failures occurred exclusively within the resin cement, indicating that the bond strength at the dentin–cement interface exceeded the internal cohesive strength of the luting resin cement [37]. This finding is consistent with the SBS results, as the SB+Dentin group demonstrated higher bond strength values than the SB+Composite group. Al-Salehi et al. [38] reported that an increased incidence of cohesive and mixed failures is associated with improved bond strength. Although no significant difference was observed between HF etching and SB in the composite groups, the lower SBS values and the predominance of adhesive failures in the SB+Composite group versus the SB+Dentin group suggest that SB of the CAD/CAM material in composite elevated cases should be interpreted with caution. The dominant fracture type remained adhesive failure at the dentin/composite–resin cement interface, while the absence of failure at the CAD/CAM–resin cement interface indicated more effective bonding of the resin cement to the CAD/CAM material than to dentin or composite substrates.

The limitations of the present study include its in vitro design, which may not fully reflect dynamic intraoral conditions, such as saliva, pH fluctuations, and repetitive occlusal forces. Mechanical aging procedures, such as chewing simulation, could not be performed due to the geometry of the CAD/CAM cylindrical samples. Notably, this study focused on midcoronal dentin surfaces, which may not fully represent the bond strength of restorative materials on deeper dentin substrates. Thus, future studies could evaluate bond strengths on deeper dentin surfaces, particularly in endocrown restorations of root canal–treated teeth. In such studies, exposing restorations to chewing simulation and evaluating microtensile bond strength in addition to thermal aging would provide more clinically relevant data. Additionally, the bonding performance of hybrid CAD/CAM blocks with different compositions should be investigated when bonded to dentin or composite substrates, and a wider range of surface pretreatment methods could be tested. Further clinical studies are recommended to validate these findings in clinical settings and optimize bonding protocols.

## 5. Conclusions

Within the limitations of this in vitro study, sandblasting (SB) and hydrofluoric acid (HF) surface pretreatments improved the shear bond strength of hybrid CAD/CAM restorations compared with untreated surfaces. Higher bond strength values were observed when bonding to dentin compared with composite substrates after surface pretreatment of the hybrid CAD/CAM material. Failure mode analysis revealed that adhesive failure at the dentin/composite–resin cement interface was the predominant failure type in most groups, whereas cohesive failure within the resin cement predominated only in the SB–dentin group. Composite elevation remains a clinically applicable approach prior to hybrid CAD/CAM restorations. The findings emphasize the critical role of surface pretreatment in achieving reliable adhesion, particularly when composite elevation is performed.

## Figures and Tables

**Table 1:** Materials used in the study. Abbreviations: UDMA, urethane-dimethacrylate; HEMA, hydroxyethyl methacrylate; BisMEPP, 2,2-bis (4-methyacryloxypolyethoxyphenyl) propane; DMA, dodecyl dimethacrylate; BISGMA, bisphenol A-glycidyl methacrylate.

Material Type	Manufacturer	Lot No.	Composition
GC Cerasmart (Nanoparticle fillers hybrid ceramic CAD/CAM block)	GC Corp. Tokyo, Japan	008671	Matrix: BisMEPP, UDMA, DMA, Filler: silica, barium glass nanoparticles (71 wt%)
RelyX Ultimate Adhesive resin cement	3M ESPE, St. Paul, MN, USA	N560996	Dimethacrylates, silane-treated fillers, initiators, stabilizers, pigments, phosphoric acid methacrylate
Gradia Direct Posterior	GC Corp, Tokyo Japan	1708182	Filler: Silica, prepolymerized fillers, fluoroaluminosilicate Glass resin matrix: UDMA, comonomer matrix
Monobond N Silane	Ivoclar Vivadent, Schaan, Liechtenstein	Z01LVG	Alcohol solution of silane methacrylate, phosphoric acid methacrylate, and sulfide methacrylate
3M Single Bond Universal Adhesive	3M ESPE, St. Paul, MN, USA	M02AV	HEMA, BISGMA, 2-propenoic acid, 2-methyl, reaction products with 1,10-decanediol and phosphorous oxide, ethanol, water, 2-propenoic acid, 2-methyl, 3-propyl ester, reaction products with vitreous silica, copolymer of acrylic and itaconic acid, camphorquinone, dimethylaminobenzoate, ethyl methacrylate

**Table 2:** Mean shear bond strength SBS values with standard deviations (±SD) in MPa for all tested groups. (*p* < 0.05). Surface pretreatments substrate interaction *p* = 0.041, effect size (Eta2) = 0.087. Different lowercase letters indicate significant differences between surfaces (rows).

	Hydrofluoric Acid (HF)	Sandblasting (SB)	Untreated	Control (Cohesive)	Effect Size	*p*
**Dentin**	13.41 ± 6.19 ^b^	15.42 ± 6.21 ^b^	4.09 ± 0.87 ^a^	35.14 ± 9.38 ^c^	0.357	**<0.001**
**Composite**	10.21 ± 1.95 ^b^	8.85 ± 3.02 ^b^	4.20 ± 0.86 ^a^	35.14 ± 9.38 ^c^	0.132	**0.007**
**Effect size**	0.057	0.204	0.000	-		
***p***	**0.043**	**<0.001**	0.959			

**Table 3:** According to the chi-square test, the distribution of failure modes showed a statistically significant difference among the groups (*p* < 0.001). The proportion of adhesive failures in the SB-dentin group was significantly lower than those observed in the untreated dentin, untreated composite, and HF-composite groups.

Group	Adhesive CAD/CAM–Cement	Adhesive Dentin/Composite–Cement	Cohesive	Mixed	*p* (Chi-Square Test)
**HF/Composite**	0	13	0	2	***p*** **< 0.001**
**HF/Dentin**	0	11	0	4	
**SB/Composite**	0	9	1	5	
**SB/Dentin**	0	4	6	5	
**Untreated/Composite**	0	14	1	0	
**Untreated/Dentin**	0	14	1	0	

## Data Availability

The original contributions presented in this study are included in the article. Further inquiries can be directed to the corresponding author.
